# Effects of Silver Fir (*Abies alba* Mill.) Needle Extract Produced via Hydrodynamic Cavitation on Seed Germination

**DOI:** 10.3390/plants10071399

**Published:** 2021-07-08

**Authors:** Francesca Ugolini, Alfonso Crisci, Lorenzo Albanese, Gabriele Cencetti, Anita Maienza, Marco Michelozzi, Federica Zabini, Francesco Meneguzzo

**Affiliations:** 1 Institute of BioEconomy—National Research Council of Italy (IBE-CNR), 10 via Madonna del Piano, Sesto Fiorentino, 50145 Firenze, Italy; alfonso.crisci@ibe.cnr.it (A.C.); lorenzo.albanese@ibe.cnr.it (L.A.); anita.maienza@ibe.cnr.it (A.M.); federica.zabini@ibe.cnr.it (F.Z.); francesco.meneguzzo@ibe.cnr.it (F.M.); 2 Institute of Biosciences and Bioresources—National Research Council of Italy (IBBR-CNR), 10 via Madonna del Piano, Sesto Fiorentino, 50145 Firenze, Italy; gabriele.cencetti@cnr.it (G.C.); marco.michelozzi@cnr.it (M.M.)

**Keywords:** *Amaranthus retroflexus* L., *Chenopodium album* L., *Conyza canadensis* L., hydrodynamic cavitation, horticultural species, natural herbicides, *Lactuca sativa* L., *Lolium perenne* L., *Pisum sativum* L., *Petroselinum crispum* (Mill.), *Solanum lycopersicum* L., weeds control

## Abstract

This paper describes the antigerminant capacity of water extracts of silver fir needles created by means of hydrodynamic cavitation processes. Fir needles (2 kg fresh weight) collected in the winter were blended and crushed in ice, poured in water only (120 L) and processed in a controlled hydrodynamic cavitation device based on a fixed Venturi-shaped reactor. The *A. alba* water extract (AWE), comprising an oil-in-water emulsion of silver fir needles’ essential oil (100% AWE), was diluted in distilled water to 75% and 50% AWE, and all aqueous solutions were tested as antigerminant against four weeds and four horticultural species and compared to control (distilled water). This study shows the effective inhibitory effect of pure AWE on germination, which mainly contains limonene (15.99 ng/mL) and α-pinene (11.87 ng/mL). Seeds showed delayed germination and inhibition but also a reduction in radicle elongation in AWE treatments as compared to control. This combined effect was particularly evident in three weeds (*C. canadensis*, *C. album* and *A. retrofllexus*) while horticultural species showed mainly effects on the radicle elongation as found in *L. sativa*, *P. crispum* and *S. lycospermum*, which showed on average 58%, 32% and 28%, respectively, shorter radicles than in the control. *P. sativum* was not affected by AWE, thus raising the hypothesis that seed characteristics and nutrition reserve might play a role in the resistance to terpenes inhibitory effect.

## 1. Introduction

In modern and intensive agriculture, weeds represent a threat for farmers as they contribute to reducing crop productivity by competing for natural resources such as light and soil nutrients [1]. To limit their presence, herbicides have been intensely spread worldwide at the expense of environment and human health. For instance, the herbicide atrazine, which was banned in many countries a few decades ago, is still found in superficial and underground water due to its high persistence within the environment [2], threatening the aquatic environment and food chain [3,4]. More recently, many controversies emerged with regard to glyphosate, the world’s most common commercial synthetic herbicide, and its several formulations [5]. It has been found to threaten the environment and human health [6,7,8,9,10,11], especially when its formulation contains surfactants like POEA (polyoxyethyleneamine) and MON 0818 (tallowamine ethoxylate), or after exposure to high doses for prolonged periods [8,12,13,14].

There is also another issue stemming from the long-term use of herbicides: in the case of glyphosate, many weeds like *Conyza* spp., *Amaranthus* spp., *Artemisia* spp. and *Lolium* spp. have developed resistance in many countries of the world [15,16].

Therefore, there is an increasing need to develop alternative and less harmful substances than synthetic herbicides for weed control and management. In this regard, the solution might be searched in the plant species that produce non-harmful secondary metabolites with allelopathic effects. The allelochemicals are secondary metabolites, by-products of the primary metabolic pathways [17], that can be produced in different plant organs (leaves, shoots, roots). Their generation is strongly affected by environmental variables such as light intensity, quality, duration, and the environmental resources and abiotic stresses [18], even though in some plants, the generation of the phenolic compounds can also be promoted by other plants, like in the case of rice defense compounds in response to the presence of barnyard grass (*Echinochloa crus-galli* L.) [19,20,21]. Allelochemicals can be volatile or enter the soil through the root exudates [22,23]. They include alkaloids, flavonoids, phenols, and glucosinolates. These molecules can impair the growth of other plants by targeting mechanisms on DNA intercalation, inhibition of DNA polymerase I and inhibition of protein biosynthesis [24], but also alters the cell’s micro- and ultra-structure, cell division and elongation, and membrane permeability [25].

In the same way as herbicides, plants allelochemicals also can be selective and can interfere the photosynthesis process at the pre- and post-emergent phase [26].

The inhibition effects on the germination of various weeds have been observed from the extracts of various species, including crops such as sorghum [27,28], sunflower and maize [29], sugar beet, safflower [30] and piper genus [31], ornamental plants such as oleander against Italian ryegrass [32] and purple nutsedge (*Cyperus rotundus* L.) [33]. Other examples count the *Tagetes* spp. (marigold), which were found to have an inhibitory effect on the germination of the hoary cress [34], *Chrysanthemum morifolium,* which inhibit other weeds [35], and wild species like *Cirsium arvense* and *Ageratum conyzoides*, which are effective against *Phalaris minor* and *Poa annua* [36].

Among trees, gymnosperms show allelopathic effects and constitute the main group in the boreal forest. In 1995, Elakovich and Wooten [37] reviewed the literature on their allelopathy and found it is mainly driven by volatile organic compounds such as terpenoids from tree leaves. For instance, in the case of silver fir (*Abies alba* L.) stands, the main volatile compounds are limonene, camphene, alpha-pinene and eucalyptol and their concentration was found remarkably dependent on different micro-ecological conditions [38]. Moreover, not only leaves are able to generate volatile compounds—twigs, seeds and cone scales also are rich in terpenes of different types and concentration, such as in limonene (twigs) and alpha-pinene (seeds and cone scales) [39,40]. In addition, the degradation of the needles on the ground has been found to lead to the production of phytotoxic leachate, which has been considered the cause of inhibition of new plants’ growth [41,42].

In general, these volatile compounds can be extracted as essential oils, which are concentrated hydrophobic liquids, from different parts of the plant. The extraction of essential oils is usually energy-consuming and not environmentally friendly, as the most common methods include steam distillation and solvent extraction. Lately, hydrodynamic cavitation has been successfully applied to the green, effective and efficient extraction of bioactive compounds, proteins, polysaccharides, flavors, fragrances, essential oils and fine chemicals (pigments and dyes) from plant materials [43,44]. 

Controlled hydrodynamic cavitation has gained a great reputation as a greener extraction method, and for its effectiveness in the intensification of food and pharmaceuticals processes [45,46]. Hydrodynamic cavitation consists of the process of generation, growth, and collapse of vapor-filled microbubbles in a liquid, at temperatures below the boiling point. During the implosion of bubbles, extremely reactive microenvironments (hot spots) occur that are locally characterized by very high temperatures, intense pressure waves, hydraulic jets and turbulence [47]. These are, in turn, associated with micro-pyrolysis events, and a limited generation of oxidant radicals, resulting in the intensification of various physical/chemical phenomena. These technologies outperform competing methods due to the fact that physicochemical and biological reactions and processes can be carried out faster and more efficiently at ambient conditions, and with lower cost. In fact, an intense energy delivery at the molecular level occurs in the course of bubbles collapse [46]. Consequently, hydrodynamic cavitation processing times are generally shorter than in conventional extraction techniques due to the maximization of the mass transfer coefficient generated by cavitation, translating into lower energy consumption [48]. Besides the increase in process yields afforded by hydrodynamic cavitation processes, either operating as a single operation unit or integrated with other technologies and processes, the same processes comply with the principles of green chemistry and the green and sustainable extraction methods of natural products [49].

Furthermore, the cheapness, straightforward scalability and superior process yields [50] are coupled with the hydrodynamic cavitation (HC) capacity to produce ultra-stable nanoemulsions [49]. Lately, research has focused on the creation of stable aqueous nano and microemulsions through the use of commercially available surfactants [51].

The objective of this study was to test the efficacy of an *A. alba* needle-water extract (AWE) produced by hydrodynamic cavitation [49] in inhibiting the germination of seeds, assessing the potential for weed control and the effects on horticultural species.

## 2. Materials and Methods

### 2.1. Plant Materials to Test AWE on Germination

AWE was assessed as an inhibitor of germination against four weeds and four horticultural species.

The choice of the invasive weed species for two reasons: the availability of seeds at the time of the experiment and their moderate requirements for germination. The seeds of the species *Chenopodium album* L. (Chenopodiaceae), *Amaranthus retroflexus* L. (Amaranthaceae), *Conyza canadensis* L. (Asteraceae) were collected at the end of the summer 2019, from mature plants growing in a non-crop area of the Pistoia province, Italy (43°56′ N 10°55′ E). The seeds were stored dry in plastic bottles at room temperature for five months until the experiments took place. *Lolium perenne* L. (Poaceae) was purchased at a local store, as it is commonly used in parks and gardens for greening lawns.

*C. album* is native to Asia, and is cultivated in some Asian countries, while in Europe and US it is considered a weed in crop fields. *A. retroflexus* is a widespread weed, native to the tropical Americas, while *C. canadensis* is native to North America and found as the first weed resistant to glyphosate [52]. In contrast, *L. perenne* is native to southern Europe, the Middle East, North Africa and eastwards to central Asia, but it has spread to other continents for many purposes, from soil stabilization to greening lawns and golf courses and in other countries where it has been introduced, it is considered an invasive species that competes with native plants [53].

AWE was also tested on seeds of horticultural species such as *Lactuca sativa* L., *Solanum lycopersicum* L. (cherry tomato), *Pisum sativum* L. and *Petroselinum crispum* (Mill.) Fuss., which can be sown in pots or in fields. The seeds of horticultural species were purchased in commercial packets.

### 2.2. Manufacturing of A. alba Needles Water Extract (AWE) by Hydrodynamic Cavitation

In early 2020, about 20 kg of *A. alba* shoots were harvested from the lowest branches of trees randomly selected in a fir forest located in the mountains of the Pistoia province (44°03′51″ N, 10°48′37″ E) in Northern Tuscany (Italy), at about 1100 m a.s.l. The shoots were placed in black plastic bags to avoid excessive water loss and brought to the laboratory within 2 h, where they were kept in a refrigerated room at 4 °C for ten days until the manufacturing of the water extract. Several samples of fir shoots, randomly taken from the storing bags, were taken out from the refrigerator and their needles detached by hand, placed in plastic bags until reaching 2 kg and kept refrigerated at 4 °C. As the first step to manufacture the *A. alba* needles water extract (AWE), needles were ground together with ice cubes by using a blender, then the mixture was processed in water (120 L), with a needle concentration of 1.67% (wt%, fresh weight). Apart from the different concentration of fir needles, the hydrodynamic cavitation process was performed as in a previous work [49] with the following details: initial temperature of the mixture of 28 °C, no heating control during the process, final temperature of the mixture of 47 °C, process time of 35 min and consumed electricity of 3.3 kWh.

### 2.3. Characterization of A. alba Needles and Water Extract (AWE)

The terpene composition of the AWE, along with raw fir needles and solid residues separated from the extract, were analyzed by gas chromatography and mass spectrometry. Regarding raw fir needles and solid residues, 1.5 g fresh weight of leaf tissue, was placed in a glass vial with 3 mL of heptane. Regarding AWE, 0.5 mL of each sample were mixed with 0.5 mL of heptane. Each vial was sealed with a Teflon septum and crimped with an aluminum cap and then vortex-mixed for five minutes, sonicated for 30 min and kept on overnight rotary agitation at 25 °C. After centrifugation at 4000 rpm for ten minutes, the heptane phase was collected for the GC-MS analysis. 

An Agilent 7820 Gas Chromatograph system equipped with a 5977E MSD with EI ionization was employed, all from Agilent Tech. (Palo Alto, AC, USA). One µL of extract in solvent was injected in a split/splitless injector operating in splitless. A Gerstel MPS2 XL autosampler equipped with a liquid option was used. The chromatographic settings were as follows: injector in splitless mode set at 260 °C, J&W innovax column (50 m, 0.20 mm i.d., 0.4 µm df); oven temperature program: initial temperature 40 °C for 1 min, then 5 °C for 1 min until 200 °C, then 10 °C for 1 min until 220 °C, then 30 °C for 1 min until 260 °C, hold time 3 min. The mass spectrometer was operating with an electron ionisation of 70 eV, in scan mode in the m/z range 29–330, at three scans sec-1. Data were acquired and analyzed using Agilent MassHunter software. The deconvoluted peak spectra, obtained by Agilent MassHunter software, were matched against NIST 11 spectral library for tentative identification. Kovats’ retention indices were calculated for further compound confirmation and compared with those reported in literature for the chromatographic column used. When available, authentic standards were also injected in order to obtain a positive identification.

AWE dilutions with distilled water (see chapter 2.3) were not analyzed, assuming that the concentrations of volatile compounds were proportional to those found in the undiluted extract.

AWE and its dilutions were assessed in terms of reaction (pH) and electric conductivity (E.C., in μS cm^−1^). These were measured using a probe with selective sensors (XS Instruments, Carpi, MO, Italy).

### 2.4. Phytotoxicity Tests of AWE

The germination test on *Lepidium sativum* L. was used as the official method for the phytotoxicity test of AWE [54]. Pure AWE (100% concentration) was compared to dilutions at two concentration levels (75% and 50%) and to the control in form of distilled water. Five petri dishes (Ø 9 cm) for each treatment (100% AWE, 75% AWE, 50% AWE) and control (CTRL) were prepared with filter paper on the bottom of the dish and placing 10 seeds of *Lepidium sativum* L. previously soaked in distilled water for one hour. All dishes were watered with 5 mL of respective solution and incubated at 22 °C for 24 h. Then, the number of germinated seeds and the radicle length was measured.

The germination index (GI, %) was calculated as”

GI(%) = (Gc × Lc) ÷ (Gt × Lt ) × 100(1)

where Gc and Gt are the average number of germinated seeds in the control and in the treatment, respectively; and Lc and Lt are the average radicle lengths of control and treatment, respectively.

### 2.5. Effects of AWE on the Germination of Weeds and Horticultural Species

For each species, 16 petri dishes (Ø 9 cm) were prepared with filter paper on the bottom of the dish. Four dishes per treatment (100% AWE, 75% AWE, 50% AWE), with the exception of *P. crispum*, with three replicates per treatment, were moistened with 5 mL of the respective solution and four more dishes with 5 mL of distilled water (CTRL). The number of seeds to place in each dish, the incubation temperature and the duration of incubation for seeds germination were set considering the findings of previous studies (Table S1). The species with temperature requirements higher than room temperature (>20 °C) were placed in the digital incubator (MyTEMP Mini, Benchmark, Sayreville NJ, USA). Seed germination was monitored every day in order to measure the radicle length (L, mm) when the seeds first showed the hypocotyl (or epicotyl in the case of peas). The number of germinated seeds (Ng) showing evident radicle (~1 mm), was recorded every day during the monitoring. The final germination rate (Gfin, %) was calculated as:

(2) [Gfin%=Ngfin÷Nt×100]

where Ngfin is the total number of germinated seeds and Nt is the total number of seeds in each petri dish.

The radicle length was measured in general by removing the seed from the petri dish and placing it next to a ruler, but in the case of *C. canadensis*, which had very short radicles, pictures were taken with a simple optical microscope with a scale as a reference, and the root length was measured by using ImageJ software 1.46r (Wayne Rasband, USA). These measurements were used to calculate the average radicle length in each petri dish.

### 2.6. Statistical Analysis

The statistical analysis was carried out in R Stat environment [55]. For each species and for each petri dish, the response was defined in the daily proportional cumulative germination curve (propCum) expressed as number of germinating seeds throughout the experiment. The “time event approach” is available in “drcSeedGerm” R packages and the specific function “makeDrm” was used to calculate the response [56].

The distributional assumption for the germination times of all the species considered was the three-parameter log-logistic (a shifted log-logistic distribution). In the code, the dose response modeling can be found at the line 49 in the “/code/modeling_drc.R” file in the repository https://github.com/alfcrisci/germination_ugolini/ (accessed on 22 June 2021).

model<- try(drm(propCum~timeBef, data = dataset, curveid = group, fct = LL2.3()))
where:
group is the replicate (petri dish)

LL2.3 is the three-parameter log-logistic (distribution) that fits the dynamics of germination times [57].

The time plots of cumulative and proportional germination curves are presented in the paper. For each fitted model the 10-percentile (T10), the median (T50) and the 90-percentile (T90) times of germination and their bounds are calculated and shown in the table. The Wald tests of estimated coefficients of logistic models were provided by using coeftest of R “lmtest” [58].

The final germination was expressed as the average percentage of the germinated seeds out of the total number of seeds between petri dishes. The final germination and the average radicle length of each petri dish in different AWE treatments were compared to control. To avoid any bias linked to the lack of both the assumption’s normality and the homogeneity of variance associated with the ANOVA model, a generalized linear model (glm) framework was prudentially adopted to perform a two-way ANOVA, considering as factors the species and the AWE treatments and control. The normality of the sample distribution was previously assessed through the Kolmogorov–Smirnov test and the homogeneity of variance was assessed through Levene median test. The data distributions are assumed to be Gaussian for the responses investigated (germination rate and length of roots), and the link of dependent variables is the data identity. The confidence interval bound (±1.96 standard deviation) was the range in which overdispersion could be considered acceptable (Table S2) The residuals plots (Fitted data vs. data residuals and Normal QQ plots) for each glm’s factorial model are available in Supplementary Materials (Figure S1). The residuals obtained from the GLM fitting are assumed with normal distribution and tested with Shapiro–Wilk normality test. Species exhibiting variance heterogeneity of germination and radicle length were excluded from further analysis, while the Tukey’s post-hoc comparison of means was finally carried out after GLM/ANOVA by using R environment by using “car” R package [59]. Data and code repository relative to the statistical analysis is available at: https://github.com/alfcrisci/germination_ugolini, accessed on 7 June 2021.

## 3. Results

### 3.1. Terpene Composition of A. alba Needles Water Extract and Other Properties

As shown in Table 1 and Figure S2, among various monoterpenes, pure AWE (100% AWE) showed the highest concentration of limonene, followed by β-pinene and camphene, while α-pinene and myrcene were present in much lower concentrations (Table 2). The total amount of monoterpenes extracted in 100% AWE per unit mass of fresh weight of fir needles was 2.37 mg/g. A complete mass balance was possible only with α-pinene and myrcene, because the concentration levels of other monoterpenes in raw fir needles, along with limonene concentration levels in the solid residues, were out of range.

Based on Table 1, it appears that the extraction yield was quite variable (37% for α-pinene and 51% for myrcene), as well as the estimated losses for α-pinene were almost double those assessed for myrcene, which prevents any reasonable estimate for the other and more abundant monoterpenes. However, it should be noted that the concentration ratio of monoterpenes in 100% AWE and solid residues was much higher for β-pinene (almost four-fold) and camphene (on average double) than for α-pinene and myrcene, which could suggest a higher extraction yield for the former monoterpenes.

Moreover, AWE dilutions were characterized by pH around 3.9 and E.C. ranging from 370 ± 2.8 (µS/cm) in 100% AWE to 204 ± 11.3 (µS/cm) in 50% AWE (280 ± 6.4 µS/cm in 75% AWE).

### 3.2. Phytotoxicity Tests of AWE

The phytotoxicity test with *L. sativum* resulted AWE as phytotoxic. In fact, germinations were observed only in CTRL (N = 8.2 ± 1.5) and in 50% AWE (N = 1.4 ± 2.6), in which the germinated seeds showed a mean radicle length of 12 ± 2 mm and 6 ± 5 mm, respectively. GI was 8% in 50% AWE and 0% in 100% AWE and in 75% AWE.

### 3.3. Effects of AWE on Final Germination of Weeds and Horticultural Species

In general, weeds (with the exception of *L. perenne*) recorded lower germination rates than horticultural species even in the control.

**Figure 1:**
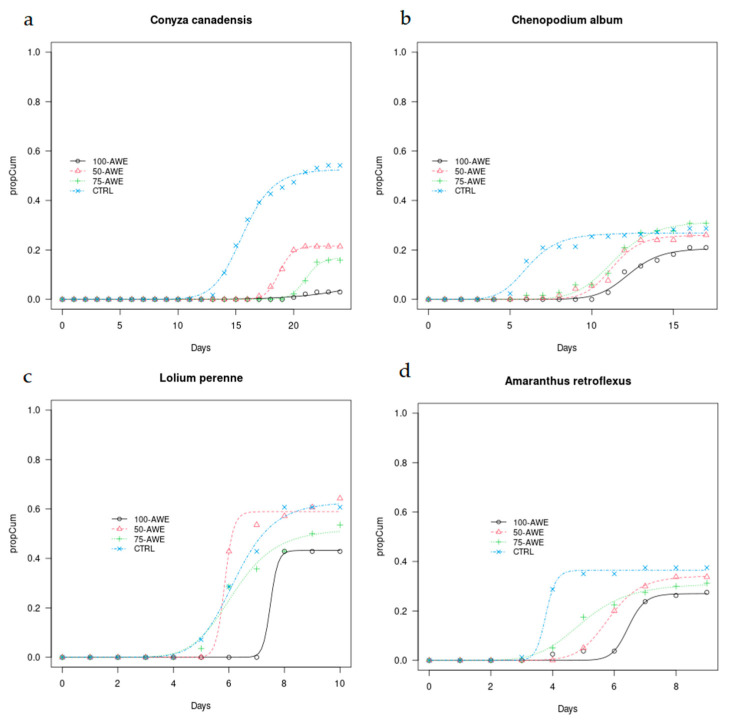
Time plots of cumulative germination curves calculated until the end of the monitoring for (**a**) *Conyza canadensis* L., (**b**) *Chenopodium album* L., (**c**) *Lolium perenne* L., (**d**) *Amaranthus retroflexus* L. under AWE treatments (50% AWE, 75% AWE, 100% AWE) and control (CTRL).

Regarding the germination models of the weeds (Figure 1), *C. canadensis* showed early germinations in CTRL (after 12 days from the beginning of the experiment). More germinations followed in the following days. In general, in CTRL and 50% AWE, the species germinated much earlier than in the treatments with higher AWE content. In 50% AWE, most germinations occurred between days 15 and 18 (when 90% of the germinated seeds emitted their root), in 75% AWE, between days 21 and 24 and in 100% AWE only a few seeds germinated in two petri dishes and mainly at day 24. Again, from the model, 10% germinations (T10) occurred 4.5, 6.7 and 11 days after CTRL in 50% AWE, 75% AWE and 100% AWE, respectively. 50% germination (T50) occurred 3, 6 and 18 days, in 50% AWE, 75% AWE and 100% AWE, respectively after CTRL and 90% germination (T90) after 2, 4 and 26 days, respectively (Table 2).

*C. album* also showed a similar pattern to *C. canadensis*, with the CTRL showing most germinations earlier than other treatments, followed by 50% AWE and 75% AWE. In 100% AWE germinations started later on, with a maximum after 15 days. However, the amount of germinated seeds varied between treatments, with the highest in CTRL (27% of total seeds) and 50% AWE (31% of total seeds) and lowest in 100% AWE (14% of total seeds). In *C. album*, T10 in AWE treatments was about 5–6 days later than CTRL, as well as 50% and 90% germination times T50 and T90 (Table 2).

*L. perenne* showed most germinations after 6 days in most treatments with the exception of 100% AWE in which germination occurred two days later. Only CTRL showed further increase of germinations in the following days while in other treatments, the increase was less marked. In *L. perenne*, T10 was only slightly affected in 50% AWE and in 100% AWE occurred 2.3 days after CTRL, while 50% germination was delayed only in 100% AWE (1.3 days). T90 occurred about at the same time in 50% AWE and 100% AWE while it is anticipated of 1.8 days in 75% AWE with respect to the control (Table 2).

Regarding *A. retroflexus*, the earliest germinations were observed in CTRL, in which 76% of the final germinations (38% seeds of the total) sprouted after 3 days. AWE dilutions and 100% AWE delayed the germination peaks by about 1–2 days. For instance, in 100% AWE most germinations occurred after 6 days and the final number of germinated seeds was 28%. In *A. retroflexus*, T10 in 50% AWE and 75% AWE was approximately the same as in CTRL while 50% germination (T50) in AWE treatments (50% AWE, 75% AWE and 100% AWE) occurred 1.2, 2 and 2.7 days respectively after CTRL, and 90% germination (T90) about 2.8 later (Table 2).

Regarding the final germination rate and radicle lengths, species exhibiting variance heterogeneity (*P. crispum* and *L. perenne* with regard to the final germination and *P. sativum* and *A. retroflexus* with regard to root length) were excluded from further analysis. For the remaining species, statistical differences were observed between species, treatments, and interaction between species and treatment (Table 3).

Regarding the weeds (Table 4), *C. canadensis* showed the maximum germination in CTRL (65% of seeds) and the minimum (2%) in 100% AWE. Germinations in AWE dilutions were also low, ranging in absolute percentages between 14% and 25% in 50% AWE and between 3% and 25% in 75% AWE. Final radicle also looked longer in CTRL although not significantly different from the AWE treatments.

*C. album* showed in general low Gfin rate, ranging around 30%, with higher rates in 50% AWE, CTRL and 75% AWE, and approximately half of that in other treatments (Gfin 14%) in 100% AWE. *C. album* showed the longest radicles in CTRL, where they measured almost double (about 11 mm) than that in the AWE treatments (5–7 mm).

*L. perenne* showed the maximum germination rate (100%) only in CTRL, while in the other treatments germination ranged between 57% (in 75% AWE and 100% AWE) and 64% (50% AWE). Regarding the radicle length, no substantial differences were found between treatments with diluted AWE, due to the high variability within the sample, while significantly longer radicles were recorded for CTRL (avg = 10.3 mm, 2.5 folds longer than in 100% AWE).

*A. retroflexus* showed similar results with longer radicles in CTRL (avg = 16 mm) and on average, 22% shorter radicles in 75% AWE (avg = 12.4 mm) and 50% shorter radicles (avg = 8 mm) in 100% AWE.

**Figure 2:**
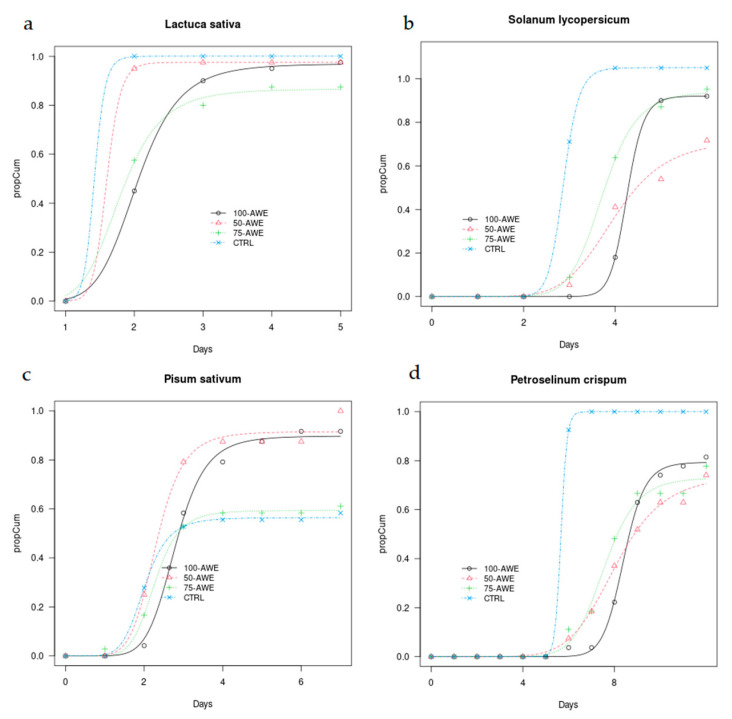
Time plots of proportional cumulative germination curves calculated until the end of the monitoring for horticultural species (**a**) *Lactuca sativa* L., (**b**) *Solanum lycopersicum* L., (**c**) *Pisum sativum* L., (**d**) *Petroselinum crispum* (Mill.) under AWE treatments (50% AWE, 75% AWE, 100% AWE) and control (CTRL).

Regarding the horticultural species (Figure 2), *L. sativa* showed similar patterns in CTRL and 50% AWE with all germinations after one day from the start of the experiment, while in the other treatments, germinations occurred around day 3. However, the final germination was high in all treatments, although slightly lower in 75% AWE. According to the model, in *L. sativa*, 10% germinations (T10) in AWE treatments were approximately at the same time of CTRL, T50 in 50% AWE, 75% AWE and 100% AWE occurred 0.4, 0.2 and 0.6 days, respectively after CTRL, while 90% germinations (T90) were 1, 0.2 and 1.2 days, respectively, later than in CTRL.

*S. lycopersicum* also started germinating in CTRL prior than in other treatments and, within two days, all seeds in this treatment germinated. In 50% AWE and 75% AWE, germinations started on average one day later and continued during the following five days, while in 100% AWE germination started later. In *S. lycopersicum*, 10% germinations in 50% AWE and 75% AWE occurred half day after CTRL and 1.4 days in 100% AWE. 50% germinations (T50) in 50% AWE, 75% AWE and 100% AWE treatments occurred 0.8, 0.9 and 1.4 days, respectively after CTRL, while 90% germinations 1.3, 2.1 and 1.4 days, respectively, later than in CTRL (Table 5).

Regarding *P. sativum*, in CTRL and AWE dilutions, it showed earlier germinations than in 100% AWE, but in this treatment as well as in 75% AWE, it showed more germinations in the following five days. In addition, *P. sativum* did not show significant differences between treatments in the final number of germinated seeds, ranging from 83% in the diluted AWE to 92% in 100% AWE. In this species, T10 was in AWE treatments was 0.3-0.7 days delayed as well as T50 and T90, although in 100% AWE, T90 occurred only one day after CTRL (Table 5).

In *P. crispum*, all seeds in CTRL germinated at day 6 while in the other treatments, germination occurred in the following days. Final germination was slightly lower in AWE treatments (74% in 100% AWE and 78% in diluted AWE) without a significant difference as compared to control. In this species, T10 in 50% AWE, 75% AWE and 100% AWE occurred 0.8, 0.6 and 1.9 days, respectively after CTRL. In addition, T50 occurred later in AWE treatments (1.9, 2.3 and 2.7 days, respectively) as well as T90 (3.3, 4.7 and 3.5 days, respectively) (Table 5).

Regarding the final germination rate and radicle length of horticultural species (Table 4), *L. sativa* showed high germination in all treatments, especially in the CTRL. The longest radicles were measured in CTRL and the shortest in 75% AWE and 100% AWE (67% and 55% shorter than radicles in the control, respectively). *P. sativum* did not show differences between treatments for any parameters, whereas *S. lycopersicum* and *P. crispum* showed similar final germination rates in all treatments, although shorter radicles were recorded in AWE treatments (on average 28% and 32% shorter than in the control, respectively).

## 4. Discussion

Comprehensive reviews are available covering the use of essential oils as herbicidal substances [51], along with other natural active ingredients [60]. The results of this study show that the water extract of *A. alba* needles produced via hydrodynamic cavitation has an inhibitory effect on the germination of most weed species, in comparison to the control. Firstly, we observed a delay in the germination of most weed species under AWE treatments, with some specificities: *C. canadensis* and *C. album* were the species that showed marked delays even at the time of 10% germinations (T10) and at T50; the latter is traditionally used in germination tests, while *L. perenne* and *A. retroflexus* were especially affected when treated with the pure water extract (100% AWE). In fact, a suppressing effect of germination was definitely observed when the AWE was used, pure which reduced germination by 97%, 48%, 43% and 27% with respect to control in *C. canadensis*, *C. album*, *L. perenne* and *A. retroflexus*, respectively. At a lower concentration, i.e., 75% AWE, germination was reduced by 77% and 43% in *C. canadensis* and *L. perenne* while only 14% and 17% reductions were observed in *C. album* and *A. retroflexus*, respectively, in comparison to control. Greater dilution, i.e., 50% AWE, had different effects on germination, depending on the species. Indeed *C. canadensis* and *L. perenne* were the most affected with 71% and 36% germination reduction, respectively.

Another effect of the AWE concerned the root elongation. Shorter roots were observed in treatments with pure AWE, which were not due to a slower growing process but to the inhibition of the root growth, as the final length was taken in all treatments when the hypocotyl (and epicotyl in peas) was formed. As seedling vigor is also important on the establishment of the future plant, impairing its growth will influence growth and plant productivity. In 100% AWE, the roots were mid-length on average as compared to the roots in the control, and *C. canadensis* and *C. album* seemed sensitive also in the treatment with 75% AWE.

Most tested horticultural species with the same extract concentrations showed only a slight delay in germination as compared to the control, with T10 and T50 occurring about half-day later. This was especially found in *L. sativa* and *P. sativum*, while *S. lycopersicum* and *P. crispum* seemed to be a little bit more affected in terms of germination times with longer time (about one day) needed to reach the reference percentages of germination. However, the final germination rates comparable to control, although they showed shorter roots in AWE treatments as compared to control, with the exception of *P. sativum*. We can hypothesize that AWE may have an inhibitory effect on the mobilization of seed reserve substances and on cell division at root level, which drive root elongation. *P. sativum* was the species least affected by AWE among all those considered, which might be linked to its seed morphology with the massive endosperm and coat.

The allelopathic effect of AWE has been attributed by many authors to the terpenes, and in this study, AWE was characterized especially by the presence of limonene and α-pinene, which are monoterpenes known for their phytotoxic effects [61,62,63,64,65]. Several studies have demonstrated the herbicidal activity of monoterpenes, in particular that of oxygenated components [65,66,67]. Abrahim et al. [61] (2000) found that the inhibition of monoterpenes on seed germination is strictly connected to their water solubility and relatively more lipophilic monoterpenes (e.g., α-pinene and limonene) had less activity than the more water-soluble oxygenated monoterpenes (e.g., camphor) in inhibiting seed germination and/or primary root growth, although they increase the oxidative metabolism of mitochondria. 

In fact, [68] found that in maize, α-pinene inhibits respiration both in the absence (basal respiration) and presence of ADP (coupled respiration), while limonene at 0.1 mM or above, stimulates basal respiration but inhibits the coupled respiration. Monoterpenes are known to alter cellular activities that require the metabolic energy of ATP and/or membrane integrity. If mitochondria are altered, seed germination and seedling growth are affected as at this stage ATP production depends mainly on mitochondrial metabolism. Indeed, α-pinene acts on mitochondria as an inhibitor of electron flow through cytochrome-oxidase pathway and this likely leads to an increase in mitochondrial reactive oxygen species and consequently, to membrane lipoperoxidation [68]. In addition, this study also found that limonene, myrcene and α-pinene significantly reduced the seedling root growth of *C. album* and inhibited seed germination.

The phytotoxic effects against various species include anatomical and physiological changes in plant seedlings, probably due to the accumulation of lipid globules in cytoplasm, reduction in mitochondria and nuclei and disruption of their membranes, inhibition of DNA synthesis in root apical meristem and a reduction of cell division [65,69,70]. Furthermore, in a different study, the content of chlorophyll was found to be lower in plants treated with monoterpenes as compared to control [63,64].

In conclusion, the urge to improve sustainability in farming practices and reduce the presence of weeds such as *C. canadensis*, *C. album* and *A. retroflexus* in cultivated areas, should foster the use of non-synthesis and less harmful products for the environment with inhibitory action on germination and the growth of weeds without affecting the crop productions [71]. The *A. alba* water extract produced via hydrodynamic cavitation seems to be a possible solution to control some of the tested weeds i.e., *C. canadensis*, *C. album*, delaying germination and inhibiting root growth, while demonstrating the limited effect on horticultural species like *P. sativum* and *S. Lycospermum*, which showed no effect on germination or a relatively lower effect on root development at higher concentration.

In addition, in other studies the water extract produced via hydrodynamic cavitation with only 1.67% fir needle concentration (fresh weight), demonstrated to be not significantly impacting on the environment due to the low persistence of essential oils-based biopesticides, as volatile compounds are easily degraded when exposed to environmental factors [72]. Beyond the low concentration level of raw fir needles, the concentration of monoterpenes in the AWE was limited due to non-negligible losses and moderate extraction yields, with substantial amounts of monoterpenes retrieved in the solid residues, which could be due to the hydrophobic nature of monoterpenes. Methods could be elaborated in order to enhance the extraction yields (e.g., adding natural emulsifiers and/or exploring other cavitation regimes), as well as to stabilize the extract (e.g., encapsulation, micro- and nano-encapsulation, nanoemulsions), allowing to control the release process. The latter is indeed a requisite to improve the stability and protect active compounds against light, oxygen and temperature, thus improving the availability and efficacy of essential oils and, at the same time, potentiate their activity [73,74,75,76,77,78,79,80].

For the sake of comparison, at least with other green methods of extraction of silver fir needles (i.e., using water as the only solvent), process yield figures can be estimated. Such estimates could be based on the consumed electricity (3.3 kWh) and either on the amount of raw fir needles in the process (2 kg) or, for example, on the total amount of monoterpenes extracted in 100% AWE per unit mass of raw fir needles (2.37 mg/g). In the first case, the process yield can be estimated at the level of 1.65 kWh/kg (consumed electricity per unit mass of raw fir needles); however, this figure could be easily increased with a higher concentration of raw fir needles in the process. In the second case, the process yield can be estimated at the level of 1.39 kWh/mg/g (consumed electricity per unit mass of extracted monoterpenes per unit mass of raw fir needles), which is approximately independent of the concentration of raw fir needles in the process, assuming that such concentration does not affect the extraction yield. Further experiments are required to check the possibility to upscale the concentration of raw fir needles in the process.

Our study was limited to laboratory experiments based on seed growth in petri dishes in controlled environments, whereas the effects of the AWE should also be tested in the real environment where multiple factors play a role on germination and growth and possibly also on AWE (e.g., light, soil, water, fertilizers). Thus, more complex experiments are recommended that aim at testing the interactions with such variables and to fully assess the efficacy of the silver fir-based water extract and the environmental effects on the ecology of the soil system.

## 5. Conclusions

This study has shown the potential of *Abies alba* L. water extract produced via hydrodynamic cavitation as an inhibitory product against germination and seedling growth of weeds that are widespread in agricultural fields. On the one hand, the pure extract has the capacity to inhibit seed germination and radicle elongation. This coupled effect was particularly evident in three weeds (*C. canadensis*, *C. album* and *A. retrofllexus*), while horticultural species showed similar effects on their radicle elongation as found in *L. sativa*, *P. crispum* and *S. lycospermum*, which showed on average 58% on 32% and 28% shorter radicles than in the control. On the other hand, *P. sativum* was not affected by AWE, suggesting that seed characteristics and nutrition reserves might play the main role in the resistance to terpenes effects on seedling growth. In conclusion, additional investigations are needed to provide a better understanding of any interactions between AWE produced via hydrodynamic cavitation and plants, to be able to assess any effects on different plant growth stages or/and in combination with other experimental variables to simulate the application in real environments (i.e., the interaction of AWE doses with soil and environmental variables etc.).

## Figures and Tables

**Table 1:** Terpene composition of the water extract obtained by hydrodynamic cavitation, raw fir needles and solid residues.

	α-Pinene	Camphene	β-Pinene	Myrcene	Limonene
100% AWE ^a^	11.87 ng/mL 712 ng/g	10.56 ng/mL 634 ng/g	0.69 ng/mL 42 ng/g	0.38 ng/mL 23 ng/g	15.99 ng/mL 959 ng/g
Raw fir needles ^b^			114 ng/g	45 ng/g	
Extraction yield ^c^			37%	51%	
Solid residues ^d^	177 ng/g	237 ng/g	37 ng/g	15 ng/g	
100% AWE/Solid residues ^e^	402%	268%	112%	153%	
Losses ^f^			31%	16%	

^a^ Concentration in 100% AWE (ng/mL) and referred to the unit mass of fir needles (ng/g, fresh weight). ^b^ Concentration in the unit mass of raw fir needles (ng/g, fresh weight). ^c^ Fraction of the monoterpenes contained in the raw fir needles released in the water extract. ^d^ Concentration in the unit mass of solid residues (ng/g, fresh weight). ^e^ Ratio of monoterpenes concentration in 100% AWE to concentration in solid residues, referred to the unit mass of fir needles (fresh weight). ^f^ Estimated losses (missing concentration of monoterpenes in 100% AWE plus solid residues, compared to raw fir needles), likely due to volatilization during the process.

**Table 2:** Estimated times of germination (10-percentile (T10), median (T50) and 90–percentile (T90)) and their bounds (Lower-Upper) for *Conyza canadensis* L., *Chenopodium album* L., *Lolium perenne* L., *Amaranthus retroflexus* L. under AWE treatments (50% AWE, 75% AWE, 100% AWE) and control (CTRL).

	*C. canadensis*			*C. album*		
	Estimate	Lower	Upper	Estimate	Lower	Upper
100% AWE: T10	24	NaN	NaN	10.3	9.34	11.43
100% AWE: T50	33.1	NaN	NaN	12.3	11.37	13.30
100% AWE: T90	45.8	NaN	NaN	14.6	12.14	17.65
75% AWE: T10	19.7	18.54	20.87	9	8.06	10.13
75% AWE: T50	21.1	20.34	21.78	11.4	10.80	11.99
75% AWE: T90	22.5	20.85	24.32	14.3	12.29	16.71
50% AWE: T10	17.5	16.60	18.51	9.5	8.39	10.86
50% AWE: T50	18.8	18.27	19.25	11.3	10.78	11.79
50% AWE: T90	20	18.98	21.22	13.3	11.68	15.20
CTRL: T10	13	12.53	13.53	4.4	3.70	5.22
CTRL: T50	15.6	15.26	15.99	6.1	5.61	6.68
CTRL: T90	18.7	17.69	19.84	8.5	6.65	10.93
	***L. perenne***			***A. retroflexus***		
	**Estimate**	**Lower**	**Upper**	**Estimate**	**Lower**	**Upper**
100% AWE: T10	7.2	6.30	8.24	5.9	5.26	6.62
100% AWE: T50	7.5	6.67	8.43	6.5	5.93	7.03
100% AWE: T90	7.8	6.79	8.95	7	6.08	8.22
75% AWE: T10	4.7	3.94	5.58	4.8	4.08	5.67
75% AWE: T50	6.2	5.39	7.06	5.8	5.35	6.35
75% AWE: T90	8.1	5.84	11.28	7.1	5.78	8.66
50% AWE: T10	5.5	NaN	NaN	3.6	2.77	4.60
50% AWE: T50	5.8	NaN	NaN	5	4.23	5.89
50% AWE: T90	6.2	NaN	NaN	7	4.63	10.50
CTRL: T10	4.9	4.23	5.59	3.4	NaN	NaN
CTRL: T50	6.2	5.73	6.76	3.8	NaN	NaN
CTRL: T90	8	6.59	9.65	4.2	NaN	NaN

**Table 3:** ANOVA of the generalized linear model (glm) between species and treatments and their interaction, for the final germination rate and the root length.

*Germination Rate*	Chi Square Value	Degree of Freedom	*p*-Value
Species	3580	5	<2.2 × 10^−16^
Treatment	75	3	3.685 × 10^−16^
Species * Treatment	275	15	<2.2 × 10^−16^
*Root length*			
Species	668.78	5	<2.2 × 10^−16^
Treatment	230.15	3	<2.2 × 10^−16^
Species * Treatment	91.35	15	5.559 × 10^−13^

**Table 4:** Final germination rate (Gfin, %), average radicle length (mm) of weeds and horticultural species in the control (CTRL) and in AWE treatments at 50%, 75% and 100%.

	*Species*	Treatment	G_fin_ (%)	Average Radicle Length (mm)
*Weeds*	*Conyza canadensis* L.	CTRL	65 ± 7 a	4.1 ± 0.3
		50% AWE	19 ± 5 b	3.3 ± 1
		75% AWE	15 ± 10 bc	2.9 ± 0.8
		100% AWE	2 ± 3 c	2.6
	*Chenopodium album* L.	CTRL	27 ± 9 ab	11 ± 1.5 a
		50% AWE	31 ± 4 a	7.3 ± 1.4 b
		75% AWE	25 ± 9 ab	5.7 ± 0.9 b
		100% AWE	14 ± 4 b	5.2 ± 1.1 b
	*Lolium perenne* L.	CTRL	100	10.3 ± 1.3 a
		50% AWE	64 ± 27	10.3 ± 1.4 ab
		75% AWE	57 ± 20	8.8 ± 3 ab
		100% AWE	57 ± 26	3.9 ± 1.3 b
	*Amaranthus retroflexus* L.	CTRL	38 ± 12 a	16 ± 2.4
		50% AWE	34 ± 17 a	12.9 ± 1.5
		75% AWE	31 ± 18 a	12.4 ± 1.3
		100% AWE	28 ± 15 a	8.3 ± 0.5
*Horticultural species*	*Lactuca sativa* L.	CTRL	100	15.6 ± 0.9 a
		50% AWE	98 ± 5	7.5 ± 0.3 ab
		75% AWE	88 ± 5	5.1 ± 0.5 c
		100% AWE	98 ± 5	7 ± 2 b
	*Pisum sativum* L.	CTRL	88 ± 8	25.5 ± 7.9
		50% AWE	83 ± 0	27.5 ± 7.3
		75% AWE	83 ± 0	23.9 ± 0.5
		100% AWE	92 ± 17	28.4 ± 7.5
	*Solanum lycopersicum* L.	CTRL	100	18.5 ± 1.7 a
		50% AWE	100	13.8 ± 1.8 b
		75% AWE	100	12.7 ± 0.7 b
		100% AWE	100	13.4 ± 1.5 b
	*Petroselinum crispum* (Mill.)	CTRL	100	14.9±1.5 a
		50% AWE	74 ± 17	10.3 ± 0.5 b
		75% AWE	78 ± 29	10.3 ± 0.9 b
		100% AWE	78 ± 11	9.9 ± 1.19 b

Mean values ± standard deviations are reported. Means followed by a common lowercase letter are not significantly different by the HSD test at 5% level of significance (HSD = Tukey’s honestly significant difference at 5% level of significance).

**Table 5:** Estimated times of germination (10-percentile (T10), median (T50) and 90-percentile (T90)) and their bounds (Lower-Upper) for *Lactuca sativa* L., *Solanum lycopersicum* L., *Pisum sativum* L., *Petroselinum crispum* (Mill.) under AWE treatments (50% AWE, 75% AWE, 100% AWE) and control (CTRL).

	*L. sativa*			*S. lycopersicum*		
	Estimate	Lower	Upper	Estimate	Lower	Upper
100% AWE: T10	1.5	1.15	1.88	3.9	3.73	4.01
100% AWE: T50	2	1.90	2.20	4.3	4.01	4.51
100% AWE: T90	2.8	2.12	3.80	4.7	4.06	5.39
75% AWE: T10	1.4	0.13	14.70	2.9	2.57	3.27
75% AWE: T50	1.6	0.37	6.94	4	3.56	4.43
75% AWE: T90	1.8	0.99	3.38	5.4	4.15	7.11
50% AWE: T10	1.3	0.87	1.81	3	2.75	3.26
50% AWE: T50	1.8	1.62	2.01	3.7	3.58	3.88
50% AWE: T90	2.6	1.97	3.37	4.6	4.24	5.08
CTRL: T10	1.3	0.20	7.84	2.5	1.66	3.86
CTRL: T50	1.4	0.25	8.13	2.9	2.58	3.19
CTRL: T90	1.6	0.25	10.34	3.3	2.64	4.05
	***P. sativum***			***P. crispum***		
	**Estimate**	**Lower**	**Upper**	**Estimate**	**Lower**	**Upper**
100% AWE: T10	2.1	1.76	2.44	7. 5	6.92	8.01
100% AWE: T50	2.8	2.62	2.99	8.4	8.09	8.72
100% AWE: T90	3.8	3.19	4.45	9.5	8.66	10.38
75% AWE: T10	1.7	1.45	1.91	6	5.28	6.88
75% AWE: T50	2.3	2.13	2.50	8	7.28	8.89
75% AWE: T90	3.2	2.65	3.85	10.7	8.40	13.71
50% AWE: T10	1.7	1.38	2.05	6.2	5.41	7.02
50% AWE: T50	2.3	2.02	2.55	7.6	7.14	8.01
50% AWE: T90	3.1	2.31	4.06	9.3	7.94	10.86
CTRL: T10	1.4	0.95	2.19	5.4	4.02	7.22
CTRL: T50	2	1.80	2.25	5.7	4.83	6.64
CTRL: T90	2.8	1.84	4.26	6	5.68	6.24

## Data Availability

https://github.com/alfcrisci/germination_ugolini (accessed on 22 June 2021).

## Supplementary Materials

The following are available online at https://www.mdpi.com/article/10.3390/plants10071399/s1, Figure S1: Gas chromatography for the terpene composition. Figure S2. Gas chromatography for the terpene composition. Table S1: Germination requirements from bibliography and settings in the experiments of this study. Table S2. Statistical summary of the final germination (upper part) and root length (lower part) for each species..
