# Supplementary material for: Effects of Silver Fir (Abies alba Mill.) Needle Extract Produced via Hydrodynamic Cavitation on Seed Germination
Source: Plants (Basel). 2021 Jul 8;10(7):1399. doi: 10.3390/plants10071399 (PMC8309281; doi:10.3390/plants10071399)
Supplement: Supplementary file 1 [file plants-10-01399-s001.zip › plants-1217814-SI.pdf]

Table S1. Germination requirements from bibliography and settings in the experiments of this study.

Species		*Chenopodium album L.*	*Amaranthus retroflexus L.*	*Conyza canadensis L.*	*Lolium perenne* L.
Total number of seeds in Petri dish (N)	CTRL	47±6	20±0	42±6	7±0
	50%AWE	53±7	20±0	52±4	7±0
	75%AWE	55±15	20±0	33±3	7±0
	100%AWE	59±12	20±0	48±1	7±0
Germination temperature (°C)		27	27	20	20
Photoperiod (hours of dark)		24	24	14	24
References		[81,83]	[82]	[84]	[85]
Monitoring duration		18	5	24	24
Interval of germination monitoring (i) (days)		1	1	1	1

Species		*Lactuca sativa* L.,	*Solanum lycopersicum* L.	*Pisum sativum* L.	*Petroselinum crispum* (Mill.) Fuss.
Total number of seeds in Petri dish (N)	CTRL	10±0	6±0	6±0	9±0
	50%AWE	10±0	6±0	6±0	7±2
	75%AWE	10±0	6±0	6±0	7±3
	100%AWE	10±0	6±0	6±0	7±1
Germination temperature (°C)		20	20	20	20
Photoperiod (hours of dark)		12	12	12	12
References		Package instructions	Package instructions	Package instructions	Package instructions
Monitoring duration		4	7	7	12
Interval of germination monitoring (i) (days)		1	1	1	1

Table S2. Statistical summary of the final germination (upper part) and root length (lower part) for each species.

**Final germination**	**Valid N**	**Mean**	**Min**	**Max**	**Standard Deviation (SD)**	**Confidence SD (-1.96)**	**Confidence SD (+1.96)**	**Coef. Var.**
C. canadensis	16	0.25	0.0	0.7	0.255	0.188	0.395	101.3
A. retroflexus	16	0.33	0.1	0.5	0.125	0.092	0.194	38.5
C. album	16	0.23	0.1	0.4	0.086	0.065	0.129	36.9
L. perenne	16	0.66	0.3	1.0	0.238	0.176	0.369	36.1
L. sativa	16	0.96	0.8	1.0	0.063	0.046	0.097	6.6
S. lycopersicum	16	1.00	1.0	1.0	0.000	0.000	0.000	0.0
P. crispum	12	0.81	0.4	1.0	0.202	0.143	0.342	25.0
P. sativum	16	0.86	0.7	1.0	0.091	0.067	0.140	10.5
**Root length (mm)**								
C. canadensis	16	2.72	0.0	4.7	1.547	1.143	2.394	56.9
A. retroflexus	16	11.98	7.7	19.1	3.060	2.261	4.736	25.5
C. album	16	7.35	4.1	12.3	2.657	1.994	3.983	36.1
L. perenne	16	8.39	2.3	12.0	3.259	2.407	5.043	38.8
L. sativa	16	9.11	4.7	16.7	4.245	3.136	6.570	46.6

S. lycopersicum	16	14.80	11.4	21.5	2.779	2.085	4.166	18.8
P. crispum	12	11.30	8.5	17.4	2.578	1.826	4.377	22.8
P. sativum	16	29.12	23.8	38.8	4.828	3.567	7.473	16.6

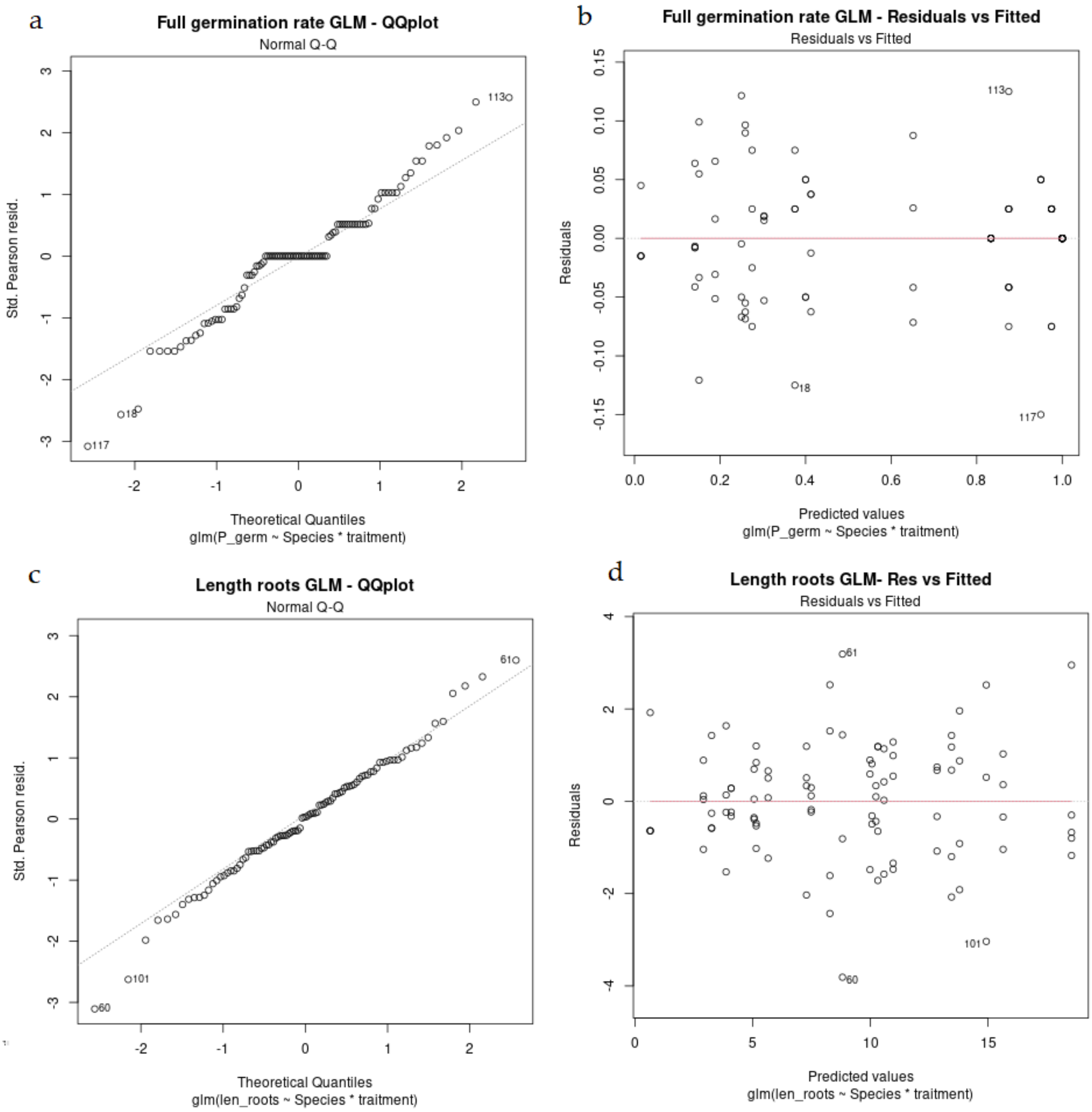

Figure S1. Plots of quantile-quantile distribution and residuals vs. fitted of generalized linear model for the final germination rate (a-b) and the root length (c-d).

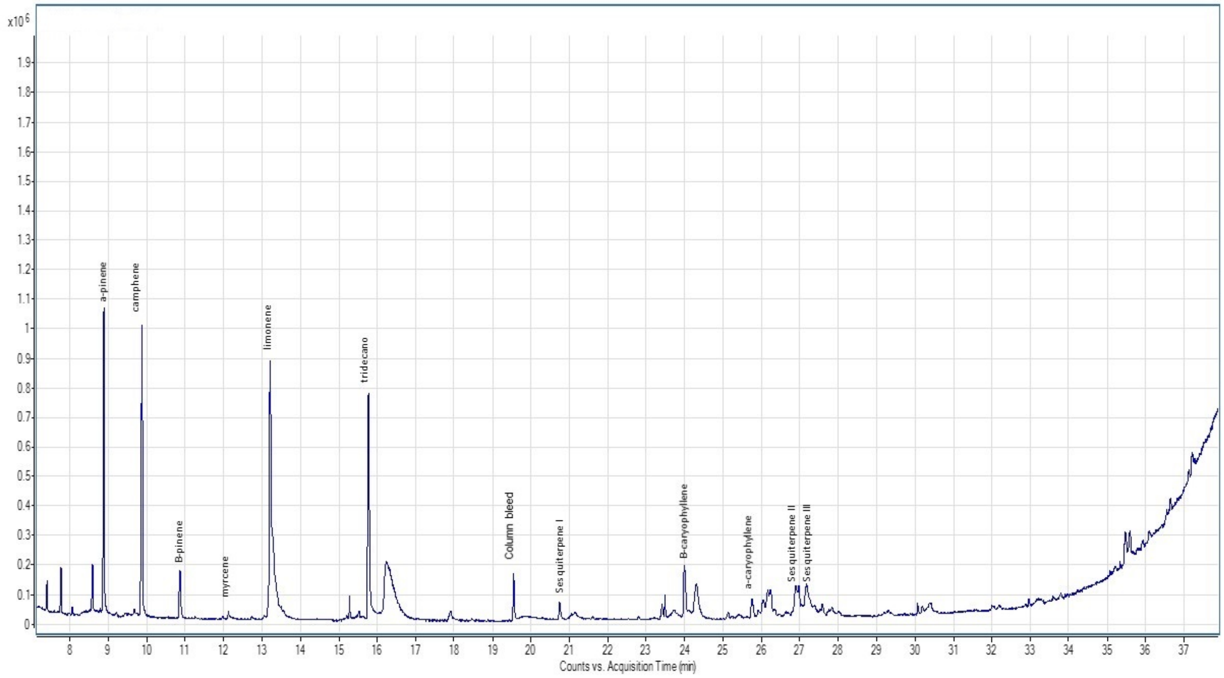

Figure S2. Gas chromatography for the terpene composition.
